# Utilisation of antibiotics in a community pharmacy: A case from north-west, South Africa

**DOI:** 10.4102/phcfm.v17i1.4943

**Published:** 2025-07-23

**Authors:** Zanele Nsingo, Varsha Bangalee, Deanne Johnston

**Affiliations:** 1 School of Health Sciences, University of KwaZulu-Natal, Durban, South Africa

**Keywords:** antibiotic, antibiotic utilisation, community pharmacy, private healthcare, antimicrobial stewardship, AWaRe classification, dispensing patterns, medicine usage

## Abstract

**Background:**

Antibiotic utilisation is a growing public health issue due to antimicrobial resistance. Community pharmacies are a key access point for antibiotics; thus, an evaluation of dispensing records will provide insights into their use.

**Aim:**

To describe the utilisation of antibiotics in a private community pharmacy.

**Setting:**

This study was undertaken in a private pharmacy located in the North West province of South Africa.

**Methods:**

A retrospective, cross-sectional study reviewed electronic dispensing records of oral antibiotics from January 2022 to August 2024, categorising them according to the World Health Organizations (WHO) Access, Watch and Reserve categories, generic status, diagnosis and payment methods.

**Results:**

A total of 10 468 antibiotic dispensing records were analysed. Adults (18–64 years) accounted for the majority of prescriptions (80.7%; *n* = 8446). Overall, Access antibiotics were mostly dispensed (56.5%; *n* = 5910); however, azithromycin, a Watch antibiotic, was the most dispensed antibiotic (*n* = 1849). Notably, 82% (*n*= 8584) of prescriptions were linked to non-specific International Classification of Diseases, 10th Revision codes. Generic medicines constituted 92.6% (*n* = 9694) of prescriptions. Although most patients (72.8%) used medical aid, cash-paying patients were more likely to be dispensed a generic antibiotic.

**Conclusion:**

Antibiotic prescribing largely aligned with WHO guidelines; however, the high rate of Access antibiotics dispensed highlights the need for targeted interventions to improve prescribing practices and guideline adherence.

**Contribution:**

This case study indicates that dispensing records contribute to improved understanding of local antibiotic usage patterns that can help combat antimicrobial resistance within a community.

## Introduction

The irrational use of antibiotics is a global challenge, with developing countries like South Africa being particularly vulnerable. One of the major consequences of irrational antibiotic use is the rise in antimicrobial resistance (AMR), which significantly contributes to increased morbidity, mortality and healthcare costs.^1^ The emergence of resistant bacteria has become a critical barrier to treating infectious diseases worldwide, with projections suggesting that if antibiotic use and awareness do not improve by 2050, bacterial infections could become the leading cause of death.^2^ The growing danger posed by antibiotic resistance has highlighted the need for awareness not only among healthcare professionals but also among patients, to ensure the rational use of antibiotics.

To address this issue, antibiotic utilisation studies play a vital role in assessing patterns of use and identifying areas for improvement. The World Health Organization (WHO) has developed the *Access, Watch*, and *Reserve* (AWaRe) classification system to guide the appropriate use of antibiotics and mitigate AMR.^2^ The *Access* category includes essential antibiotics for treating common infections with a lower risk of resistance such as amoxicillin and doxycycline. The goal is for at least 60% of all antibiotics prescribed to come from the *Access* group (WHO).^2^ The *Watch* category includes antibiotics with a higher resistance risk, which should be used cautiously for specific infections such as azithromycin and ciprofloxacin. The *Reserve* category consists of ‘last-resort’ antibiotics like linezolid, which are critical for treating multidrug-resistant infections and should be used only when necessary.^2^

Misdiagnosis and empirical prescribing without proper diagnostic confirmation contribute to the misuse of antibiotics, accelerating the development of AMR. A study analysing antibiotic prescribing among South African general practitioners found that 45.4% of antibiotic prescriptions were inappropriate, highlighting the need for improved diagnostic accuracy and stewardship.^3^ To enhance diagnostic precision and ensure appropriate prescribing, healthcare providers utilise standardised coding systems. The International Classification of Diseases, 10th Revision (ICD-10), is a globally recognised system for coding diseases, conditions and medical procedures. In South Africa, the use of ICD-10 codes is integral to studying antibiotic use because these codes provide a standardised framework for documenting diagnoses and health conditions. This ensures accuracy in reporting, which is essential for categorising antibiotic prescriptions and is according to the AWaRe framework.^4^ By linking specific ICD-10 codes to diagnoses, it is possible to assess how antibiotics are being used for different health conditions and identify patterns of misuse, such as overprescribing for conditions where antibiotics are not warranted. The use of ICD-10 codes also supports national efforts to standardise health data, track disease prevalence and inform policies aimed at improving antibiotic use.^5^

The public healthcare sector in South Africa has made progress in promoting antimicrobial stewardship programmes, which are designed to improve antibiotic prescribing and reduce misuse. These programmes emphasise adherence to evidence-based Standard Treatment Guidelines, monitoring antibiotic use and educating healthcare providers on the risks of overprescribing.^3^ However, such initiatives are not as widespread or robust in the private sector, where adherence to Standard Treatment Guidelines is not mandatory and individual prescriber discretion plays a larger role in treatment decisions.^6^ While the private sector offers more resources and treatment options, it lacks the structured oversight and enforceable regulations found in the public sector, making antibiotic prescribing more susceptible to variation and misuse,^3^ further fuelling AMR.^7^

Antibiotic utilisation studies are essential for gaining a comprehensive understanding of prescribing practices in real-world healthcare settings. By examining these patterns, such studies can help identify areas where improvements are needed.^8^ In community pharmacies, where antibiotics are frequently dispensed, the prescribing practices and adherence to treatment guidelines are crucial for combating AMR.^8^

This study aimed to explore antibiotic prescribing practices in a private community pharmacy in the north-west province of South Africa. Specifically, it sought to identify the commonly prescribed antibiotics, the conditions for which they were prescribed and dosage forms. The study also examined whether these prescriptions, along with their associated ICD-10 codes, were aligned with the recommendations outlined in the South African Standard Treatment Guidelines (STGs), Primary Health care (PHC) Level. The STGs are the national guidelines developed by the South African Department of Health to ensure equitable access to healthcare, and promote rational use of medicines by ensuring that the medicines provided are cost-effective, appropriate and aligned with the burden of diseases in South Africa.^9^ The STGs relate to the Essential Medicines List (EML). The medicines on the EML are the most necessary medicines to meet the priority needs of the population. The South African EML aligns with the WHO’s EML, which provides a global standard for medicines selection according to safety, efficacy and cost-effectiveness.^9^ This alignment is important because it ensures that the prescribing practices at the Primary Health care or outpatients are aligned with both national and global standard policies to exercise good antimicrobial stewardship practices. Furthermore, seasonal variations in prescribing patterns and methods of payment such as medical aid or out-of-pocket payments were also investigated. Examining these factors is intended to provide valuable insights that could help promote rational antibiotic use and inform interventions to improve antibiotic stewardship in community pharmacy settings.

## Research methods and design

### Study design

This study was a quantitative descriptive retrospective, cross-sectional drug utilisation study. The study reviewed electronic prescriptions for oral antibiotics that were dispensed by the pharmacy from 01 January 2022 to 31 August 2024.

### Setting

The study was conducted in a community pharmacy in a town in the north-west province of South Africa. The population of this town is less than 50 000 and is surrounded by numerous towns and villages. Many residents rely on the town for essential services, including healthcare, education and shopping. The pharmacy in question offers a wide range of services including dispensing of prescriptions and over-the-counter medicines and basic health screens through the clinic such as blood pressure and blood glucose monitoring. The pharmacy also plays a vital role in facilitating access to Primary Health care in an area where public health facilities are often overburdened.

### Study population and sampling strategy

All patients, adults and children, who received oral antibiotics during the study period from January 2022 to August 2024, were included. A total of 10 468 prescriptions were analysed. Records were excluded if the antibiotics were not administered orally as well as if there were incomplete patient records, for example, missing demographic data.

### Data collection

De-identified prescription data were extracted by the gatekeeper from their dispensing software and were provided to the researchers in a Microsoft® Excel spreadsheet. The variables extracted included the date when the prescription was dispensed, patient gender, date of birth, whether the dispensed antibiotic was the originator brand or generic, product description, dosage form, strength, pack size, ICD-10 code, payment method (cash or medical aid) and levy charged.

### Data analysis

The extracted data underwent coding and cleaning to remove incomplete records, duplicates and any prescriptions that did not meet the inclusion criteria. The cleaned dataset was then exported to Stata version 18 for analysis, by a statistician. All antibiotics were categorised according to the WHO’s AWaRe classification system which divides antibiotics into three: *Access, Watch* and *Reserve*.^2^

The dataset was numerical in nature, where the overall counts of patients receiving antibiotics were determined according to specific parameters. Descriptive statistics were used to summarise the data, with results presented as frequencies and percentages. To assess associations between patient characteristics (such as age group and payment method) and the likelihood of generic medicine use, Inferential Statistical Methods were applied. Specifically, logistic regression analysis was used to estimate odds ratios (ORs) with 95% confidence intervals (CIs) and *p*-values were reported to determine statistical significance, with *p* < 0.05 considered significant.

### Ethical considerations

Prior to the commencement of the study, gatekeeper approval was obtained from the owner of the pharmacy. Ethical clearance to conduct this study was obtained from the University of KwaZulu-Natal’s Biomedical Research Ethics Committee, (No. BREC/00006684/2024). The data received from the gatekeeper were de-identified, neither patient names nor identification numbers were provided; no information was provided relating to the prescriber.

## Results

The original dataset consisted of 15 014 antibiotics dispensed between 01 January 2022 and 30 August 2024. After removing records that did not meet the inclusion criteria, a total of 10 468 dispensing records were assessed.

### Overview of participant demographics

The prescriptions comprised 1.6% (*n* = 165) of patients less than 2 years of age, 8.5% (*n* = 889) were between the ages of 3 and 11 years, 2.8% (*n* = 294) were between the ages of 12 and 17 years, 80.7% (*n* = 8446) were between the ages of 18 and 64 years, and 6.4% (*n* = 674) of the prescriptions were for patients who were 65 years and above. Just more than half the antibiotics dispensed were to female patients (53.5%; *n* = 5596).

Table 1 presents the number of antibiotics dispensed in the study, categorised according to the WHO’s AWaRe Classification System (*Access, Watch* and *Reserve*), and stratified by age group and gender.

**TABLE 1:** Overview of antibiotics dispensed according to age and gender in percentages (%).

Antibiotic	Age group (years)					Gender	
	< 2 (*n* = 165)	2–11 (*n* = 889)	12–17 (*n* = 294)	18–64 (*n* = 8446)	≥ 65 (*n* = 674)	Male (*n* = 5596)	Female (*n* = 4870)
Azithromycin (*n* = 1849) (Watch)	1.6	8.8	2.7	82.0	5.1	54.2	45.9
Clavulanic acid and amoxicillin (*n* = 1814) (*Access*)	1.6	12.0	3.8	76.8	5.9	59.2	40.8
Amoxicillin (*n* = 1490) (*Access*)	2.4	10.0	3.2	80.6	3.9	45.1	54.9
Metronidazole (*n* = 1182) (*Access*)	0.3	4.2	1.9	89.3	4.4	46.3	53.7
Cefuroxime (*n* = 866) (*Watch*)	2.5	14.7	5.2	71.9	5.7	63.1	37.0
Ciprofloxacin (*n* = 641) (*Watch*)	0.3	1.1	1.0	83.3	14.4	52.9	47.1
Cefpodoxime (*n* = 537) (*Watch*)	5.8	18.8	2.6	66.7	6.2	57.7	42.3
Co-trimoxazole (*n* = 508) (*Access*)	1.6	6.7	0.6	84.4	6.7	42.5	57.5
Doxycycline (*n* = 315) (*Access*)	0.0	0.3	1.6	93.0	5.1	51.8	48.3
Fosfomycin (*n* = 259) (*Access*)	0.0	0.4	3.1	84.2	12.4	60.2	39.8
Clarithromycin (*n* = 257) (*Watch*)	0.0	0.4	1.2	85.6	12.9	56.4	43.9
Kanamycin (*n* = 199) (*Watch*)	2.5	15.6	5.0	71.9	5.0	62.8	37.2
Flucloxacillin and amoxicillin (*n* = 107) (*Watch*)	0.1	2.8	3.7	87.9	4.7	51.4	48.6
Clindamycin (*n* = 102) (*Access*)	0.0	0.0	2.0	81.4	16.7	57.9	42.2
Nitrofurantoin (*n* = 76) (*Access*)	0.0	0.0	1.3	75.0	23.7	60.5	39.5
Dapsone (*n* = 48) (*Access*)	0.0	0.0	0.0	100.0	0.0	58.3	41.7
Ampicillin and cloxacillin (*n* = 34) (*Access*)	0.0	0.0	0.0	85.3	14.7	38.2	61.8
Lymecycline (*n* = 33) (*Access*)	0.0	0.0	3.0	84.9	12.1	78.8	21.2
Cefixime (*n* = 31) (*Watch*)	0.0	3.2	0.0	96.7	0.0	38.7	61.3
Nitrofurantoin (*n* = 30) (*Access*)	0.0	10.0	0.0	76.7	13.3	53.3	46.7
Erythromycin (*n* = 28) (*Watch*)	0.0	3.6	7.1	78.6	10.7	60.7	39.3
Moxifloxacin (*n* = 24) (*Watch*)	0.0	0.0	0.0	79.2	20.8	75.0	25.0
Flucloxacillin (*n* = 19) (*Access*)	0.0	10.5	10.5	73.7	5.3	36.8	63.2
Levofloxacillin (*n* = 19) (*Watch*)	0.0	0.0	0.0	89.5	10.5	31.6	68.4

In this study, the top 60% of prescribed antibiotics were azithromycin (17.7%), amoxicillin/clavulanic acid (17.3%), amoxicillin (14.2%) and metronidazole (11.3%). Most of the antibiotics were from the *Access* category 54.5% (*n* = 5910), while the remaining 43.5% (*n* = 4558) were from the *Watch* category. There were no antibiotics dispensed from the *Reserve* category.

Reviewing the commonly prescribed *Watch* category antibiotics in greater detail found that azithromycin (17.7%) was the most prescribed. It was further observed that ciprofloxacin was also commonly prescribed (6.1%), of which eight prescriptions were under the ICD-10 code A49.9 for unspecified bacterial infections.

Upon review of antibiotics used in paediatric patients, it was observed a few antibiotics were dispensed despite these typically being avoided in this population. There were 15 patients under the age of 18 years, who received ciprofloxacin. Two patients, between the ages of 2 and 11 years, were prescribed doxycycline and lymecycline.

### Indication of antibiotics

All antibiotics dispensed had a correlating ICD-10 code. A total of 200 ICD-10 codes were identified within the dataset. The majority of prescriptions (82%; *n* = 8565) dispensed were associated with Z codes, which generally describe health encounters or conditions not tied to specific diseases (e.g., routine exams, preventive care). A total of 17 different Z codes were recorded. The remaining 18% (*n* = 1884) were associated with non-Z ICD-10 codes. Of these, prescriptions with human immunodeficiency virus (HIV)-related ICD-10 code (B24) were excluded for further analysis (*n* = 203, 1.9%), as they typically reflect prophylactic or therapeutic use of antibiotics for opportunistic infections in HIV-positive patients. Within this excluded group, cotrimoxazole (*n* = 192) and dapsone (*n* = 8) were the most frequently prescribed antibiotics. From the remaining prescriptions, the 12 most frequently prescribed codes were presented in Table 2 and were selected for detailed analysis. These were chosen as they provided a cross-sectional view of the primary clinical reasons for antibiotic prescribing beyond the Z-related and HIV-related codes.

**TABLE 2:** Antibiotics (%) used to treat the top 12 diagnoses.

Antibiotic	J20.9^a^ (*n* = 224)	A49.9^b^ (*n* = 151)	J10.1^c^ (*n* = 78)	R52.9^d^ (*n* = 59)	A09.9^e^ (*n* = 54)	J11.1^f^ (*n* = 48)	N39.0^g^ (*n* = 39)	J03.3^h^ (*n* = 36)	J18^i^ (*n* = 35)	J22^j^ (*n* = 35)	J00^k^ (*n* = 34)	N30.0^l^ (*n* = 33)
Amoxicillin (*Access*)	1.3	11.9	0.0	40.7	0.0	4.2	0.0	0.0	11.4	2.9	17.7	0.0
Ampicillin and cloxacillin (*Access*)	0.0	11.9	0.0	0.0	0.0	0.0	0.0	0.0	2.9	0.0	0.0	0.0
Azithromycin (*Watch*)	61.2	0.0	78.2	5.1	5.6	31.3	2.6	13.9	25.7	65.7	35.3	0.0
Cefixime (*Watch*)	0.0	0.0	1.28	0.0	0.0	0.0	0.0	0.0	2.9	2.9	0.0	0.0
Cefpodoxime (*Watch*)	1.8	5.3	0.0	1.7	0.0	18.8	0.0	2.8	11.4	0.0	5.9	0.0
Cefuroxime (*Watch*)	21.4	15.2	14.1	1.7	1.9	2.1	2.6	75	2.9	22.9	5.9	6.1
Ciprofloxacin (*Watch*)	0.0	5.3	1.3	0.0	22.2	0.0	35.9	0.0	0.0	0.0	0.0	63.6
Clarithromycin (*Watch*)	8.9	0.0	1.3	0.0	0.0	2.1	0.0	8.3	2.9	0.0	0.0	0.0
Clavulanic acid and amoxicillin (*Access*)	4.5	24.5	0.0	17.0	1.9	35.4	0.0	0.0	31.4	0.0	29.4	21.2
Clindamycin (*Access*)	0.0	2.7	0.0	0.0	1.9	0.0	0.0	0.0	0.0	0.0	0.0	0.0
Co-trimoxazole (*Access*)	0.0	6.0	0.0	1.7	18.5	0.0	0.0	0.0	0.0	2.9	0.0	0.0
Doxycycline (*Access*)	0.0	6.6	0.0	3.4	0.0	0.0	5.1	0.0	2.9	0.0	5.9	0.0
Flucloxacillin (*Access*)	0.0	1.3	0.0	0.0	0.0	0.0	0.0	0.0	0.0	0.0	0.0	0.0
Fosfomycin (*Access*)	0.5	2.7	2.6	3.4	0.0	0.0	48.7	0.0	0.0	0.0	0.0	6.1
Kanamycin (*Watch*)	0.0	0.7	0.0	5.1	37.0	0.0	0.0	0.0	0.0	0.0	0.0	0.0
Metronidazole (*Access*)	0.0	5.3	1.3	18.6	11.1	4.2	5.1	0.0	5.7	0.0	0.0	0.0
Moxifloxacin (*Watch*)	0.5	0.0	0.0	0.0	0.0	0.0	0.0	0.0	0.0	2.9	0.0	0.0
Nitrofurantoin (*Access*)	0.0	0.0	0.0	1.7	0.0	2.1	0.0	0.0	0.0	0.0	0.0	3.0

a , Acute bronchitis;

b , Bacterial infection, unspecified;

c , Influenza with respiratory manifestations;

d , Pain;

e , Gastro-enteritis and colitis of unspecified origin;

f , Influenza with other manifestations;

g , Urinary tract infections, site not specified;

h , Acute Streptococcal tonsillitis;

i , Pneumonia, unspecified organism;

j , Unspecified lower respiratory infection;

k , Common cold;

l , Acute cystitis.

A review of prescriptions by ICD-10 codes revealed that the most commonly prescribed antibiotic overall was azithromycin (*Watch* category), particularly for respiratory-related diagnoses, such as influenza with respiratory manifestations (J10.1, 78.2%), unspecified acute lower respiratory infection (J22, 65.7%) and the common cold (J00, 35.3%). Cefuroxime (*Watch*) was also widely used, especially for acute *Streptococcal* tonsillitis (J03.3, 75.0%) and bacterial infections of unspecified origin (A49.9, 15.2%). Among *Access* category antibiotics, amoxicillin was the most frequently prescribed, notably for pain (R52.9, 40.7%) and the common cold (J00, 17.7%). Ciprofloxacin (*Watch*) and fosfomycin (*Access*) were predominantly used for urinary tract infections (N39.0 and N30.0), with ciprofloxacin accounting for 63.6% of prescriptions for acute cystitis (N30.0) and fosfomycin accounting for 48.7% for urinary tract infections (site not specified).

### Antibiotics prescribed per season

**FIGURE 1:**
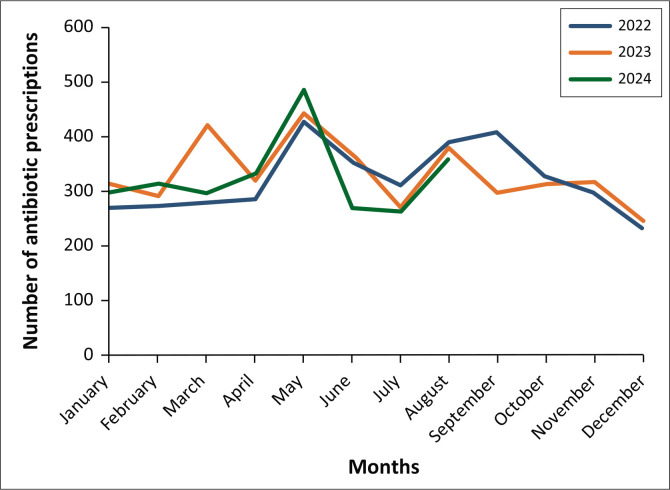
Antibiotics prescribed per season from 2022 to 2024.

Figure 1 illustrates the monthly variations in antibiotic prescriptions over the study period, highlighting seasonal trends. On average, 327.13 (range: 246–488) antibiotic prescriptions were issued per month over the study period. Peaks in antibiotics use were seen during autumn (March–May, *n* = 3298), which is 31.5% and winter (June–August, *n* = 2967), which is 28.3%. In contrast, spring (September–November) had the lowest number of prescriptions (*n* = 1960), which is 18.7%. In all the 3 years, the highest number of antibiotics were dispensed in May (average: 452; range: 427–488).

### Choice of antibiotic and payment method

There were more generic antibiotics dispensed, 92.61% (*n* = 9694) as opposed to originator brands, 7.39% (*n* = 774). Antibiotics, such as amoxicillin (*n* = 1490), co-trimoxazole (*n* = 508), doxycycline (*n* = 315), clarithromycin (*n* = 257), flucloxacillin and amoxicillin combination (*n* = 107), clindamycin (*n* = 102), erythromycin (*n* = 28), moxifloxacin (*n* = 24), flucloxacillin (*n* = 19) and levofloxacin (*n* = 19) were exclusively prescribed as generics. While only the originator brands for kanamycin (*n* = 199) and dapsone (*n* = 48) were dispensed as there is currently no generics available for those in the market. Both generic and originator brands for fosfomycin (originator, *n* = 102; generic, *n* = 157) and nitrofurantoin (originator, *n* = 62; generic, *n* = 62) were dispensed.

Both male and female patients frequently opted for generics, with male patients demonstrating a stronger preference (91.4%) for generics compared to female patients. This was statistically significant, with an odds ratio of 1.49 (95% CI: 1.29–1.74, *p* < 0.001).

Patients aged 18–64 years (OR = 1.92, 95% CI: 1.20–3.09, *p* = 0.01) and those 65 years or older (OR = 1.97, 95% CI: 1.13–3.45, *p* = 0.02) were significantly more likely to use generics compared to those less than 2 years old. There were no significant differences in generic use for age groups 2–11 years (*p* = 0.74) and 12–17 years (*p* = 0.45).

Medical aid transactions were the dominant payment method, accounting for 72.8% (*n* = 7617) of the total transactions as compared to cash patients at 27.2% (*n* = 2851). Of those transactions which were paid by medical aid, 40.0% (*n* = 3133) of these claims incurred no levy, while the highest levy paid was ZAR R1 131.74. From the patients who were paying cash, the highest amount paid was ZAR R892.83. Patients paying with cash were twice as likely to use generics compared to those on medical aid (OR = 1.90, 95% CI: 1.56–2.30, *p* < 0.001).

## Discussion

This study reviewed 10 468 dispensing records for oral antibiotics dispensed from January 2022 to 31 August 2024. The data revealed a wide age distribution of antibiotic prescriptions, with most prescriptions (80.7%) being for patients aged 18 to 64 years. Only 12.9% of prescriptions were for paediatric patients (< 18 years). This skewed distribution towards adults reflects the higher incidence of infections requiring antibiotics in this age group, potentially due to occupational exposures and lifestyle factors.

Despite the lower percentage of paediatric prescriptions, the prescribing practices in this age group warrant attention, especially regarding inappropriate prescriptions of antibiotics such as ciprofloxacin, doxycycline and lymecycline, which are contraindicated in younger patients. Ciprofloxacin, for example, is associated with the risk of musculoskeletal toxicity, particularly tendon damage, in children, which may lead to irreversible joint and cartilage damage. A study by Menschik et al.^10^ observed chondrocyte toxicity and necrosis in human adult cartilage specimens exposed to ciprofloxacin *in vitro*. While this study was conducted on adult cartilage, it raises concerns about the potential effects of ciprofloxacin on cartilage in younger populations. Similar caution has been emphasised in global guidelines and clinical research, such as a study in India that reported inappropriate prescribing of ciprofloxacin for paediatric patients despite the availability of safer alternatives.^11^ While ciprofloxacin may be indispensable for treating resistant bacterial infections, its prescription should strictly adhere to guidelines, especially for paediatric cases. The use of fluoroquinolones, such as ciprofloxacin, is generally contraindicated in children under 18 due to these potential adverse effects, especially in those who are still growing.^12^

Doxycycline is known to deposit in growing bones and teeth, leading to growth retardation, discolouration and enamel hypoplasia. Studies emphasise that tetracyclines, including doxycycline, should be avoided in children under 12 years, except in cases of severe or life-threatening infections such as rickettsial diseases or anthrax.^13^ These findings align with observations from Kenya, where the overuse of tetracyclines in paediatric cases was linked to non-adherence to age-specific prescribing guidelines.^14^

Inappropriate antibiotic use in children also contributes to the growing problem of AMR. Misuse of antibiotics for viral infections, or in situations where the benefits do not outweigh the risks, can promote the development of resistant strains of bacteria, leading to infections that are harder to treat in the future.^15^ The promotion of appropriate prescribing practices, adherence to evidence-based guidelines, and the judicious use of antibiotics are essential in preserving the efficacy of these drugs and ensuring the safety of paediatric patients.

The AWaRe classification framework highlights the necessity for prudent antibiotic use to address the global challenge of AMR.^2^ The findings of this study align with global trends, where *Access* antibiotics (56.5%) are predominantly prescribed, although this falls slightly short of the recommended 60.0% threshold set by the WHO for optimal antibiotic use in clinical practice.^2^ While antibiotics can only be dispensed upon producing a valid prescription from an authorised prescriber in South Africa, both pharmacists and prescribers play a vital role in the rational use of antibiotics and combatting AMR.^16,17^ As pharmacists are the last point of contact before antibiotics reach the patients, they are tasked with the responsibility of reviewing the prescription and counselling patients. Thus, it is essential that there is collaboration between pharmacists and prescribers to support antimicrobial stewardship initiatives by improving compliance with the STGs and ensuring the appropriate use of antibiotics.^18^

The frequent use of azithromycin (*n* = 1849), which falls within the *Watch* category, mirrors trends reported in other settings. For instance, a study in Pakistan found a similar over-reliance on macrolides like azithromycin for infections with unclear bacterial aetiology, raising concerns about resistance development.^19^ Possible drivers of this trend include a lack of diagnostic tools, habitual prescribing patterns and pressure from patients expecting antibiotics.^20,21^ To address the inappropriate use of azithromycin, targeted prescriber education and patient awareness is necessary as well as other antimicrobial stewardship programmes are essential.

Similarly, ciprofloxacin, also categorised under the *Watch* list, was frequently prescribed (eight prescriptions) for non-specific ICD-10 codes like A49.9. This reflects a broader challenge noted in countries like Vietnam, where ciprofloxacin was often prescribed without microbiological evidence.^22^ In this study, bacteriological confirmation could not be confirmed as this information was obtained from the pharmacy dispensing records, which do not capture laboratory or diagnostic data. The absence of microbiological testing and the use of non-specific ICD-10 codes suggested that most treatment decisions were based on the clinical signs and symptoms rather than laboratory evidence. This is consistent with the findings in South African community healthcare settings, where microbiological testing is seldom conducted prior to prescribing antibiotics.^23^ While ideal prescribing would involve confirmatory diagnosis from the laboratory, routine testing in community settings may not be practical because of cost, accessibility and time pressures in busy primary care environments. For example, in South Africa, the National Pathology Group Guidelines indicate that the process of performing culture and sensitivity results involves different procedures, each incurring separate charges, which cannot be affordable, especially for patients who are paying out of pocket and those with medical aids that do not cover such procedures. Therefore, while it is understandable to prescribe antibiotics based on clinical signs and symptoms, this underscores the importance of strengthening the adherence to evidence-based guidelines to combat AMR.^24^

Interestingly, no prescriptions for *Reserve* antibiotics were recorded in this study. This finding aligns with observations from other regions, such as Nepal, where *Reserve* category antibiotics like linezolid were rarely available in community pharmacies.^25^ Similarly, a study in Etitrea found that none of the prescriptions had *Reserve* category antibiotics, indicating a minimal use in that setting.^26^ Furthermore, a research conducted in Limpopo province in South Africa between 2014 and 2018 in the public sector indicated the antibiotics that were found available and the *Reserve* category were < 1%.^27^ While this study is limited to one community pharmacy in a small town, these findings collectively suggest that the use of *Reserve* antibiotics is generally low in various settings. The limited use is encouraging as it reflects the appropriate restriction of last-resort antibiotics, a key strategy in mitigating the risk of antimicrobial resistance.

Certain diagnoses, such as R52.9 (pain) and J00 (common cold), are typically not associated with bacterial infections and therefore do not require antibiotic treatment.^2^ Despite this, a significant number of antibiotic prescriptions were issued for these conditions, which may indicate potential misclassification of diagnoses or coding errors, where healthcare providers may have assigned ICD-10 codes that did not indicate a bacterial cause, but still resulted in antibiotic prescriptions.^28^ This highlights the need for a more accurate diagnosis coding and a deeper understanding of the ICD-10 codes system. Training healthcare providers in the accurate use of ICD-10 codes can help prevent unnecessary prescriptions and ensure that antibiotics are only prescribed when truly needed. For gastroenteritis and colitis, unspecified (A09.9), the most frequently prescribed antibiotics were ciprofloxacin (22.2%), metronidazole (11.1%), and co-trimoxazole (18.5%), Notably, ciprofloxacin, a *Watch* category antibiotic, was the most commonly used, despite the fact that antibiotics are not routinely indicated for A09.9 as per STGs.^9^

Conditions like *J20.9* (acute bronchitis) and *J10.1* (influenza with respiratory manifestations) are typically viral in origin, and antibiotics are generally not recommended unless a secondary bacterial infection occurs.^9^ The high number of prescriptions, particularly for azithromycin (61.2%), cefuroxime (21.4%) and clarithromycin (8.9%), suggested that antibiotics were being prescribed empirically, without clear evidence of bacterial involvement. This underscores the need for stronger antimicrobial stewardship, especially in managing viral infections.^29^ The over-prescription of antibiotics in these cases contributes significantly to antimicrobial resistance, as unnecessary antibiotic use accelerates resistance development.^2^ Efforts to raise awareness among healthcare providers about the risks of prescribing antibiotics for viral conditions are crucial for reducing AMR.

For conditions where antibiotics are warranted, for example, J18 (pneumonia, unspecified organism) showed prescriptions of amoxicillin (11.4%), azithromycin (25.7%), cefuroxime (2.9%) and clavulanic acid/amoxicillin (31.4%). For J03.3 (acute *Streptococcal* tonsillitis), the predominant antibiotics were cefuroxime (75.0%) and azithromycin (13.9%), while *Access* antibiotics like amoxicillin and doxycycline were minimally prescribed or absent. In cases of N30.0 (acute cystitis), ciprofloxacin (63.6%) and clavulanic acid/amoxicillin (21.2%) were most frequently prescribed, with lower use of *Access* antibiotics such as fosfomycin (6.1%) and nitrofurantoin (3.0%). For N39.0 (urinary tract infection, unspecified), fosfomycin accounted for 48.7% of prescriptions, followed by ciprofloxacin (35.9%) and doxycycline (5.1%). While antibiotic use is justified for these infections, the marked preference for broad-spectrum *Watch* antibiotics, such as azithromycin, cefuroxime and ciprofloxacin, over narrower-spectrum *Access* antibiotics raises concerns about adherence to STG recommendations. This also emphasises the need for improved antimicrobial stewardship to reduce the risk of antimicrobial resistance. *Access* antibiotics, such as amoxicillin and doxycycline, should be the preferred first-line treatment.^2^

The high use of *Watch* antibiotics across several diagnoses, including those that are often viral or self-limiting, suggests potential overuse and highlights the need for strengthened antimicrobial stewardship. In contrast, *Access* antibiotics were underutilised in some instances where they may have been more appropriate according to STGs. This trend raises concerns about the practices in the private healthcare sector, where prescribers often have more autonomy and can prescribe according to their discretion, rather than strictly adhering to the STGs.^30^ Such freedom may lead to variability in antibiotic choices, contributing to the overuse of broad-spectrum antibiotics and increasing the risk of AMR. With the introduction of the National Health Insurance (NHI) in South Africa, managing these prescribing practices will become crucial. The NHI’s potential to standardise treatment protocols in both public and private sectors will reduce the differences in prescribing. This shift will enforce adherence to guidelines, such as STGs and EML, across all healthcare levels and may help curb inappropriate prescribing.^30^ Ensuring consistent antibiotics stewardship will therefore be essential for the success of the NHI framework.

The dataset reveals seasonal variations in antibiotic prescriptions. This aligns with studies documenting similar seasonal trends, such as Degnan et al.,^31^ who reported increased antibiotic use in colder months due to higher respiratory infection rates and according to Alabi and Essack,^30^ who observed a correlation between seasonal variations in antibiotic prescribing and the prevalence of viral infections with bacterial complications. Similarly, Suda et al.^32^ noted lower antibiotic use in warmer months, attributed to reduced infection rates and changes in patients’ behaviour. This seasonal pattern underscores the need for monitoring and adjusting prescribing practices to mitigate unnecessary antibiotic use during peak infection periods.

The overwhelming preference for generic antibiotics (92.61%) aligns with global cost-saving trends, as generic entry into the market has been shown to influence prescribing practices. A study found that the introduction of generic ciprofloxacin led to an increase in consumption, suggesting that affordability significantly influences prescribing and dispensing patterns.^29^ Additionally, the clinical impact of generic substitution remains a key consideration, with studies emphasising the need to assess patient adherence, clinical outcomes and cost-effectiveness.^33^

Payment methods also impact the likelihood of generic antibiotic use. While medical aid was the dominant payment method (72.8%), patients paying with cash were twice as likely to use generics (OR = 1.90), likely due to cost considerations. Cash-paying patients also bore a higher financial burden, with the highest payment recorded at ZAR R892.83. This aligns with studies that highlight the economic motivations behind generic substitution and the potential for increased financial strain on patients paying out-of-pocket.^34^

Patient demographics significantly influence generic antibiotic use. Male patients were more likely to use generics than female patients (OR = 1.49), and patients aged 18–64 years and ≥ 65 years had higher odds of using generics compared to those < 2 years. This suggests that affordability and accessibility shape prescribing patterns. In contrast, younger children (< 2 years) had the lowest percentage of generic use (87.9%), most likely due to the limited availability of paediatric formulations among generics.^35,36^

The findings of this study highlight several key challenges and opportunities for improving antibiotic stewardship. The inappropriate use of antibiotics for conditions like viral infections and non-specific ICD-10 codes contributes to antimicrobial resistance and unnecessary healthcare costs.^37^ Paediatric prescribing practices require particular attention, with stricter adherence to guidelines for antibiotics such as ciprofloxacin and doxycycline.

To address these challenges, coordinated interventions are essential. These include mandatory prescription audits,^38,39^ enhanced training on AWaRe classifications^4^ and increased public awareness about the risks of antimicrobial misuse.^39^ Seasonal patterns in antibiotic prescriptions suggest opportunities for targeted public health initiatives during peak periods. Additionally, efforts to improve affordability and accessibility of antibiotics, especially for paediatric formulations, can help ensure equitable healthcare outcomes.^4^ The CDC outlines core elements of antimicrobial stewardship in hospital setting, while it is developed for the United States; this can be adapted in similar healthcare settings.^39^ A multicentre study conducted in South Africa demonstrated that a pharmacist-led antimicrobial stewardship programme across both urban and rural hospitals resulted in a significant reduction in antibiotic use by 18%.^18^ This success illustrates that antimicrobial stewardship can be effectively implemented by non-specialist healthcare professionals, including pharmacists, nurses and general practitioners, through structured interventions such as prescription audits, adherence to STGs and quality improvement processes.^18^ One of the education strategies that they were using was the ‘Train-the-Trainer’ Model which allows pharmacists to transfer skills sustainably across teams, while audits and feedback provide critical, real-time insights to prescribers.^40^ The same strategy of ‘Train-the-Trainer’ also worked in Malawian tertiary hospitals, where pharmacists were upskilled to train other healthcare workers.^41^ Similar successes have been reported in other African nations. In Kenya, an antimicrobial stewardship implemented at Kenyatta National Hospital demonstrated improvements in prescribing practices and reduced inappropriate antibiotic use, particularly after training healthcare workers and introducing clinical decision support tools.^42^ In Nigeria, pharmacist-led audits and feedback sessions significantly improved the rational use of antibiotics and compliance with institutional treatment protocols.^43^

These findings highlight the broader relevance of pharmacist-led antimicrobial stewardship programmes in LMIC settings, especially when supported by local leadership, education models such as Train-the-Trainer and regular performance feedback. Future efforts should build on these models and explore context-specific barriers and enablers to expand the reach and sustainability of stewardship programmes across African primary and community healthcare sectors.

### Limitations

This study was limited to retrospective dispensing records, where clinical outcomes or rationales for prescriptions were not assessed. Future research should consider prospective audits where clinical data and interventions could be incorporated.

Although the results provide valuable insight to the study site upon which antibiotic interventions could be built, the data may not be transferable to other private pharmacies, especially those outside the area.

## Conclusion

This study underscores the value of reviewing local dispensing records as a critical tool for informing targeted interventions to optimise antibiotic use and combat antimicrobial resistance (AMR). By analysing real-world dispensing patterns, healthcare systems can identify key areas for improvement, such as aligning prescribing practices with AWaRe Guidelines and addressing inconsistencies in paediatric antibiotic use. Strengthening the use of ICD-10 codes in prescribing can further enhance data accuracy, supporting more precise monitoring and intervention efforts. Improving adherence to these coding standards and refining paediatric prescribing practices will not only promote rational antibiotic use but also contribute to better patient outcomes and a more effective response to AMR.
