# Corrigendum: Utilisation of antibiotics in a community pharmacy: A case from North-West, South Africa

**DOI:** 10.4102/phcfm.v17i1.5273

**Published:** 2025-11-19

**Authors:** Zanele Nsingo, Varsha Bangalee, Deanne Johnston

**Affiliations:** 1 School of Health Sciences, University of KwaZulu-Natal, Durban, South Africa

In the published article, Nsingo Z, Bangalee V, Johnston D. Utilisation of antibiotics in a community pharmacy: A case from North-West, South Africa. Afr J Prm Health Care Fam Med. 2025;17(1), a4943. https://doi.org/10.4102/phcfm.v17i1.4943, on page 5, under the heading ‘Choice of antibiotic and payment method’, the following was incorrectly formulated.

Instead of:

Both generic and originator brands for fosfomycin (originator, *n* = 102; generic, *n* = 157) and nitrofurantoin (originator, *n* = 62; generic, *n* = 62) were dispensed.

It should be:

Both generic and originator brands for fosfomycin (originator, *n* = 102; generic, *n* = 157) and nitrofurantoin (originator, *n* = 62; generic, *n* = 62) were dispensed. Correct formula is (originator, *n* = 102; generic, *n* = 157) and nitrofurantoin (originator, *n* = 82; generic, *n* = 24).

On page 6, paragraph 4, there was a spelling mistake. Instead of: Etitrea, the correct spelling is: Eritrea.

On page 3, Table 1, Nitrofurantoin was listed twice and Levofloxacin was misspelt.

Instead of:

**TABLE 1:** Overview of antibiotics dispensed according to age and gender in percentages (%).

Antibiotic	Age group (years)					Gender	
	< 2 (*n* = 165)	2–11 (*n* = 889)	12–17 (*n* = 294)	18–64 (*n* = 8446)	≥ 65 (*n* = 674)	Male (*n* = 5596)	Female (*n* = 4870)
Azithromycin (*n* = 1849) (*Watch*)	1.6	8.8	2.7	82.0	5.1	54.2	45.9
Clavulanic acid and amoxicillin (*n* = 1814) (*Access*)	1.6	12.0	3.8	76.8	5.9	59.2	40.8
Amoxcillin (*n* = 1490) (*Access*)	2.4	10.0	3.2	80.6	3.9	45.1	54.9
Metronidazole (*n* = 1182) (*Access*)	0.3	4.2	1.9	89.3	4.4	46.3	53.7
Cefuroxime (*n* = 866) (*Watch*)	2.5	14.7	5.2	71.9	5.7	63.1	37.0
Ciprofloxacin (*n* = 641) (*Watch*)	0.3	1.1	1.0	83.3	14.4	52.9	47.1
Cefpodoxime (*n* = 537) (*Watch*)	5.8	18.8	2.6	66.7	6.2	57.7	42.3
Co-trimoxazole (*n* = 508) (*Access*)	1.6	6.7	0.6	84.4	6.7	42.5	57.5
Doxycycline (*n* = 315) (*Access*)	0.0	0.3	1.6	93.0	5.1	51.8	48.3
Fosfomycin (*n* = 259) (*Access*)	0.0	0.4	3.1	84.2	12.4	60.2	39.8
Clarithromycin (*n* = 257) (*Watch*)	0.0	0.4	1.2	85.6	12.9	56.4	43.9
Kanamycin (*n* = 199) (*Watch*)	2.5	15.6	5.0	71.9	5.0	62.8	37.2
Flucloxacillin and amoxicillin (*n* = 107) (*Watch*)	0.1	2.8	3.7	87.9	4.7	51.4	48.6
Clindamycin (*n* = 102) (*Access*)	0.0	0.0	2.0	81.4	16.7	57.9	42.2
Nitrofurantoin (*n* = 76) (*Access*)	0.0	0.0	1.3	75.0	23.7	60.5	39.5
Dapsone (*n* = 48) (*Access*)	0.0	0.0	0.0	100.0	0.0	58.3	41.7
Ampicillin and cloxacillin (*n* = 34) (*Access*)	0.0	0.0	0.0	85.3	14.7	38.2	61.8
Lymecycline (*n* = 33) (*Access*)	0.0	0.0	3.0	84.9	12.1	78.8	21.2
Cefixime (*n* = 31) (*Watch*)	0.0	3.2	0.0	96.7	0.0	38.7	61.3
Nitrofurantoin (*n* = 30) (*Access*)	0.0	10.0	0.0	76.7	13.3	53.3	46.7
Erythromycin (*n* = 28) (*Watch*)	0.0	3.6	7.1	78.6	10.7	60.7	39.3
Moxifloxacin (*n* = 24) (*Watch*)	0.0	0.0	0.0	79.2	20.8	75.0	25.0
Flucloxacillin (*n* = 19) (*Access*)	0.0	10.5	10.5	73.7	5.3	36.8	63.2
Levonoxacillin (*n* = 19) (*Watch*)	0.0	0.0	0.0	89.5	10.5	31.6	68.4

It should be:

**TABLE 1:** Overview of antibiotics dispensed according to age and gender in percentages (%).

Antibiotic	Age group (years)					Gender	
	< 2 (*n* = 165)	2–11 (*n* = 889)	12–17 (*n* = 294)	18–64 (*n* = 8 446)	≥ 65 (*n* = 674)	Male (*n* = 5 596)	Female (*n* = 4 870)
Azithromycin (*n* = 1849) (*Watch*)	1.6	8.8	2.7	82.0	5.1	54.2	45.9
Clavulanic acid and amoxicillin (*n* = 1 814) (*Access*)	1.6	12.0	3.8	76.8	5.9	59.2	40.8
Amoxicillin (*n* = 1 490) (*Access*)	2.4	10.0	3.2	80.6	3.9	45.1	54.9
Metronidazole (*n* = 1 182) (*Access*)	0.3	4.2	1.9	89.3	4.4	46.3	53.7
Cefuroxime (*n* = 866) (*Watch*)	2.5	14.7	5.2	71.9	5.7	63.1	37.0
Ciprofloxacin (*n* = 641) (*Watch*)	0.3	1.1	1.0	83.3	14.4	52.9	47.1
Cefpodoxime (*n* = 537) (*Watch*)	5.8	18.8	2.6	66.7	6.2	57.7	42.3
Co-trimoxazole (*n* = 508) (*Access*)	1.6	6.7	0.6	84.4	6.7	42.5	57.5
Doxycycline (*n* = 315) (*Access*)	0.0	0.3	1.6	93.0	5.1	51.8	48.3
Fosfomycin (*n* = 259) (*Access*)	0.0	0.4	3.1	84.2	12.4	60.2	39.8
Clarithromycin (*n* = 257) (*Watch*)	0.0	0.4	1.2	85.6	12.9	56.4	43.9
Kanamycin (*n* = 199) (*Watch*)	2.5	15.6	5.0	71.9	5.0	62.8	37.2
Flucloxacillin and amoxicillin (*n* = 107) (*Watch*)	0.1	2.8	3.7	87.9	4.7	51.4	48.6
Clindamycin (*n* = 102) (*Access*)	0.0	0.0	2.0	81.4	16.7	57.9	42.2
Nitrofurantoin (*n* = 106) (*Access*)	0.0	2.8	0.9	75.5	20.8	58.5	41.5
Dapsone (*n* = 48) (*Access*)	0.0	0.0	0.0	100.0	0.0	58.3	41.7
Ampicillin and cloxacillin (*n* = 34) (*Access*)	0.0	0.0	0.0	85.3	14.7	38.2	61.8
Lymecycline (*n* = 33) (*Access*)	0.0	0.0	3.0	84.9	12.1	78.8	21.2
Cefixime (*n* = 31) (*Watch*)	0.0	3.2	0.0	96.7	0.0	38.7	61.3
Erythromycin (*n* = 28) (*Watch*)	0.0	3.6	7.1	78.6	10.7	60.7	39.3
Moxifloxacin (*n* = 24) (*Watch*)	0.0	0.0	0.0	79.2	20.8	75.0	25.0
Flucloxacillin (*n* = 19) (*Access*)	0.0	10.5	10.5	73.7	5.3	36.8	63.2
Levofloxacin (*n* = 19) (*Watch*)	0.0	0.0	0.0	89.5	10.5	31.6	68.4

The author(s) apologise(s) for this error. The correction does not change the study’s findings of significance or overall interpretation of the study’s results or the scientific conclusions of the article in any way.

